# Supplementary material for: Yeast homologs of human MCUR1 regulate mitochondrial proline metabolism
Source: Nat Commun. 2020 Sep 25;11:4866. doi: 10.1038/s41467-020-18704-1 (PMC7519068; doi:10.1038/s41467-020-18704-1)
Supplement: Supplementary file 1 — Supplementary Information [file 41467_2020_18704_MOESM1_ESM.pdf]

Supplementary Information

Yeast homologs of human MCUR1 regulate mitochondrial proline metabolism

Zulkifli et al.

a

DELTA-BLAST hits of Human MCUR1 (UniProt ID:Q96AQ8-1) in *S. cerevisiae* Refseq_protein database				
**Protein**	**Query Coverage**	**E value**	**Identity**	**Accession**
Fmp32 (Put6)	54%	2e-40	25.00%	NP_116608.1
Ylr283w (Put7)	55%	2e-08	18.57%	NP_013385.1

b

DELTA-BLAST hits of *S. cerevisiae* Fmp32 (UniProt ID:P43557) in Human Refseq_protein database				
MCUR1	84%	5e-57	24.48%	NP_001026883.1
CCDC90B	84%	7e-57	21.88%	NP_068597.2
**DELTA-BLAST hits of *S. cerevisiae* Ylr283w (UniProt ID:Q05867) in Human Refseq_protein database**				
MCUR1	60%	4e-48	19.32%	NP_001026883.1
CCDC90B	52%	4e-40	16.30%	NP_068597.2

c

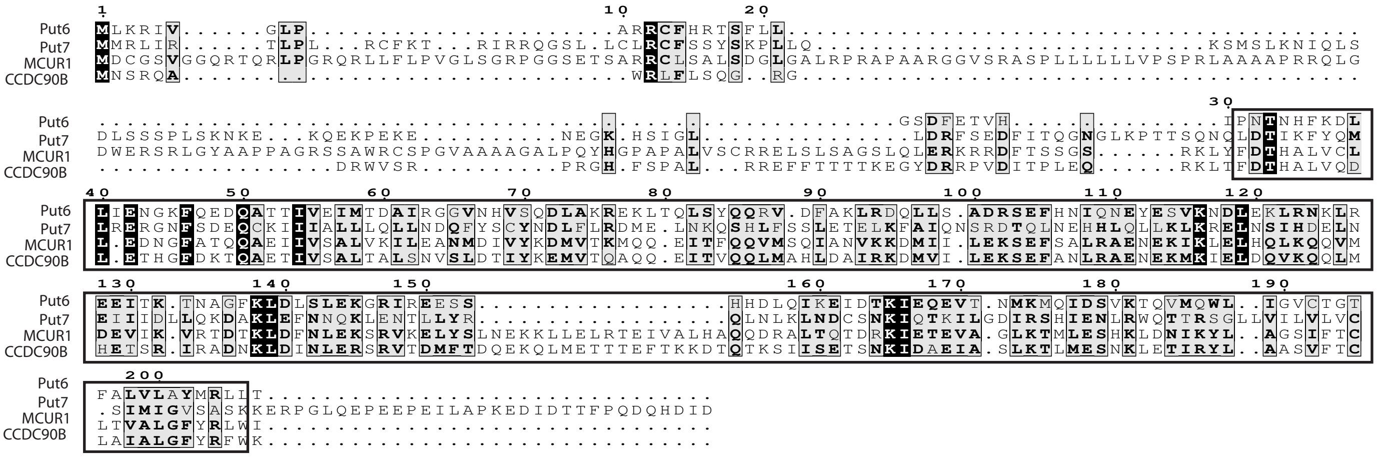

Supplementary Fig.1: Sequence analysis of MCUR1, Put6 and Put7. (a) DELTA-BLAST analysis of human MCUR1 as query with *S. cerevisiae* reference protein database. **(b)** DELTA-BLAST analysis of *S. cerevisiae* Fmp32 (Put6) and Ylr283w (Put7) protein as query with *H. sapiens* reference protein database. Top hits from the analysis are presented with % query coverage, E values, % identity and accession number. **(c)** Multiple sequence alignment of *S. cerevisiae* Put6, Put7, MCUR1 and its paralog CCDC90B showing sequence conservation in the predicted DUF1640 region (highlighted by box).

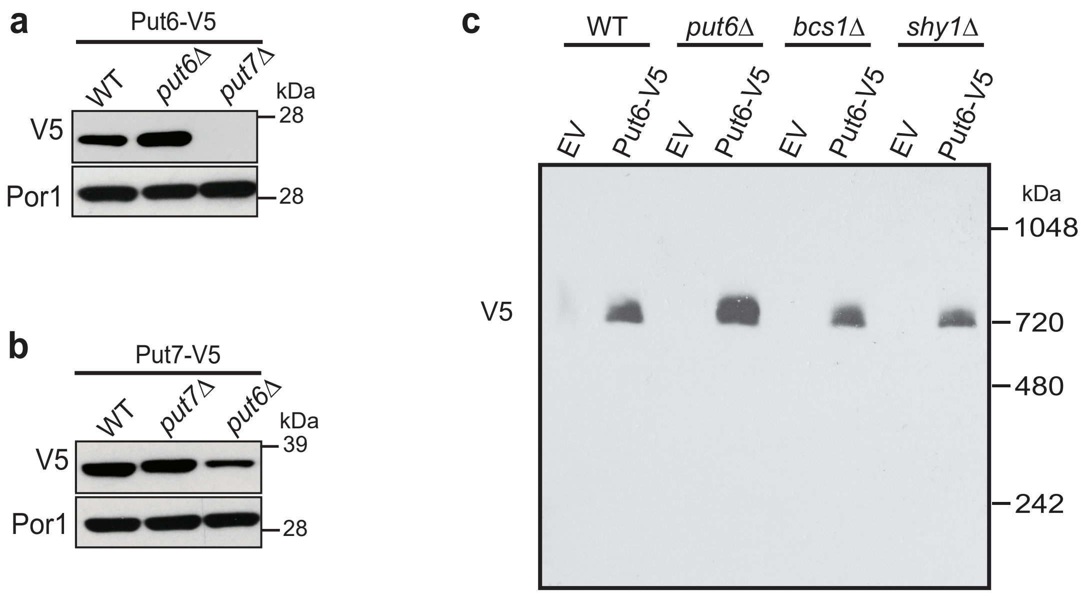

Supplementary Fig. 2: Put6 and Put7 protein levels depend on each other but not on mitochondrial respiratory chain complexes. (a and b) SDS-PAGE immunoblot analysis of mitochondrial proteins from the indicated strains expressing Put6-V5 or Put7-V5. Por1 was used as loading control. Data is representative of three biologically independent experiments. **(c)** Mitochondria from WT, *put6Δ*, *bcs1Δ*, and *shy1Δ* cells transformed with empty vector or Put6-V5 were solubilized in digitonin and analyzed by BN-PAGE immunoblotting using anti-V5 antibody. Data is representative of two biologically independent experiments. The source data are provided as Source Data file.

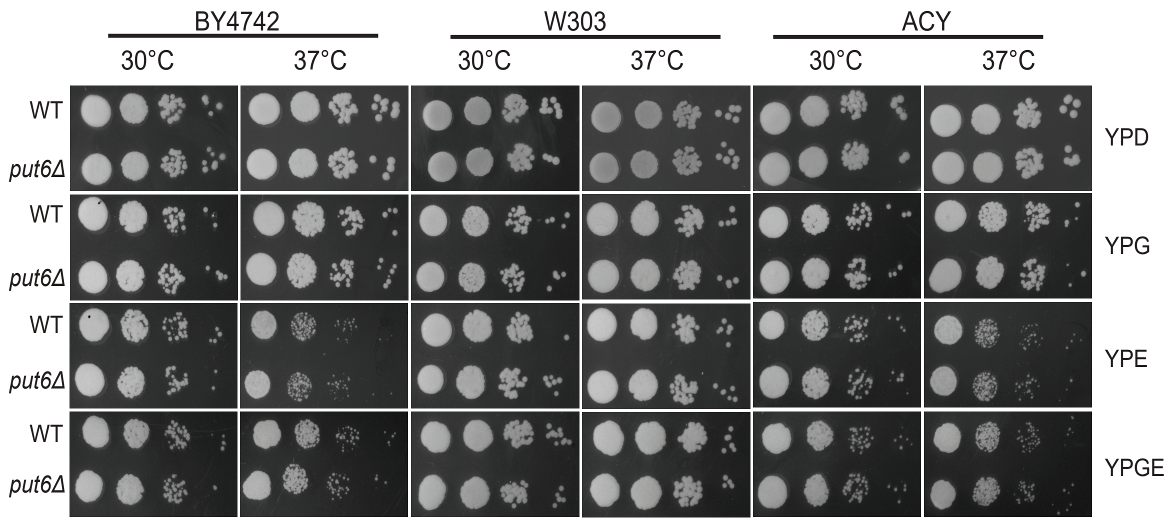

Supplementary Fig. 3: Put6 is not required for respiratory growth. Ten-fold serially diluted WT and *put6Δ* yeast cells from three different genetic backgrounds (BY4742, W303 and prototrophic yeast ACY) were seeded on YPD, YPG, YPE and YPGE plates at 30°C and 37°C. Pictures were taken after 2 days of growth in YPD or 5 days of growth in YPG, YPE, and YPGE media. Data is representative of three independent experiments.

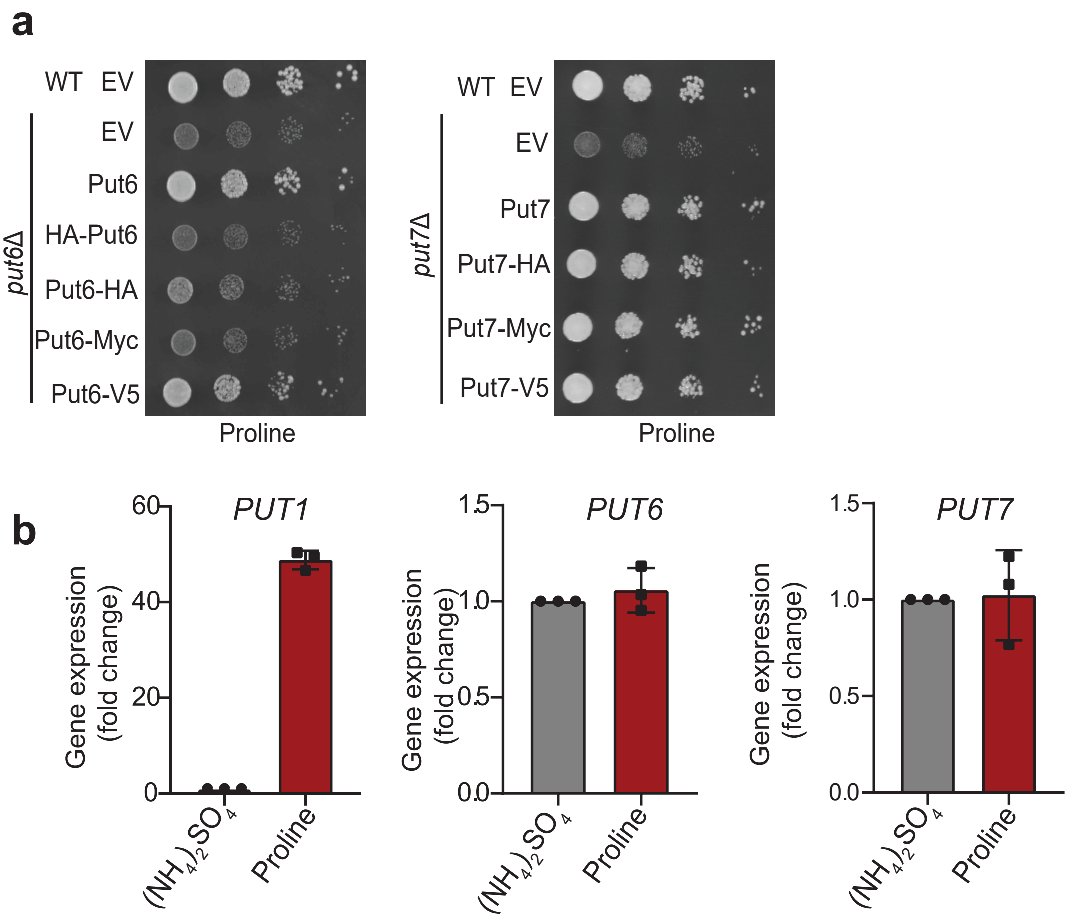

Supplementary Fig. 4: (a) Ten-fold serial dilutions of indicated yeast strains harboring empty vector (EV) or tagged versions of Put6/Put7 were seeded on synthetic media plates containing proline as the sole nitrogen source and incubated at 30°C. Pictures were taken after 3 days of seeding. All the above data is representative of atleast three biologically independent experiments. **(b)** WT yeast cells were grown in synthetic galactose-containing media with ammonium sulfate or proline as the sole nitrogen source and *PUT1*, *PUT6* and *PUT7* transcript levels were measured by qRT-PCR. *ACT1* was used as an internal control and *PUT1*, which is known to be up regulated in proline-containing media, was used as a positive control. Data are normalized to transcript levels from cells cultured in ammonium sulfate and are expressed as mean ± SD (n=3 biologically independent experiments). The source data are provided as Source Data file.

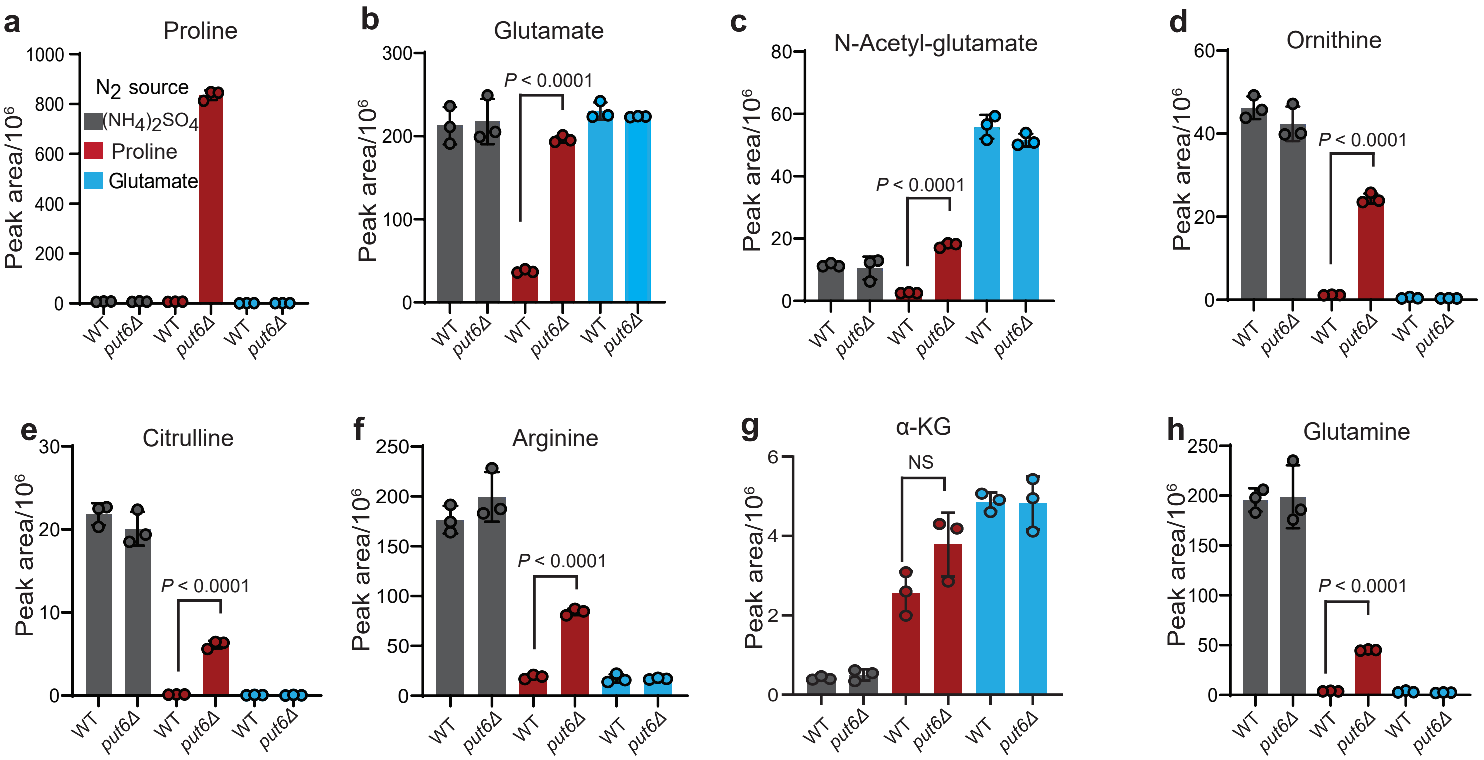

Supplementary Fig. 5: LC-MS-based quantification of (a) proline, (b) glutamate, (c) N-acetyl glutamate, (d) ornithine, (e) citrulline, (f) arginine, (g) α-ketoglutarate, and (h) glutamine in WT and *put6Δ* yeast cells cultured in ammonium sulfate (grey bars), proline (red bars) or glutamate (blue bars) as the sole nitrogen source. (Data are potted as mean ± SD, n=3 biologically independent experiments)**.** Statistical significance was assessed by one-way analysis of variance (ANOVA) with Tukey's multiple comparison test; NS- not significant. The source data are provided as Source Data file.

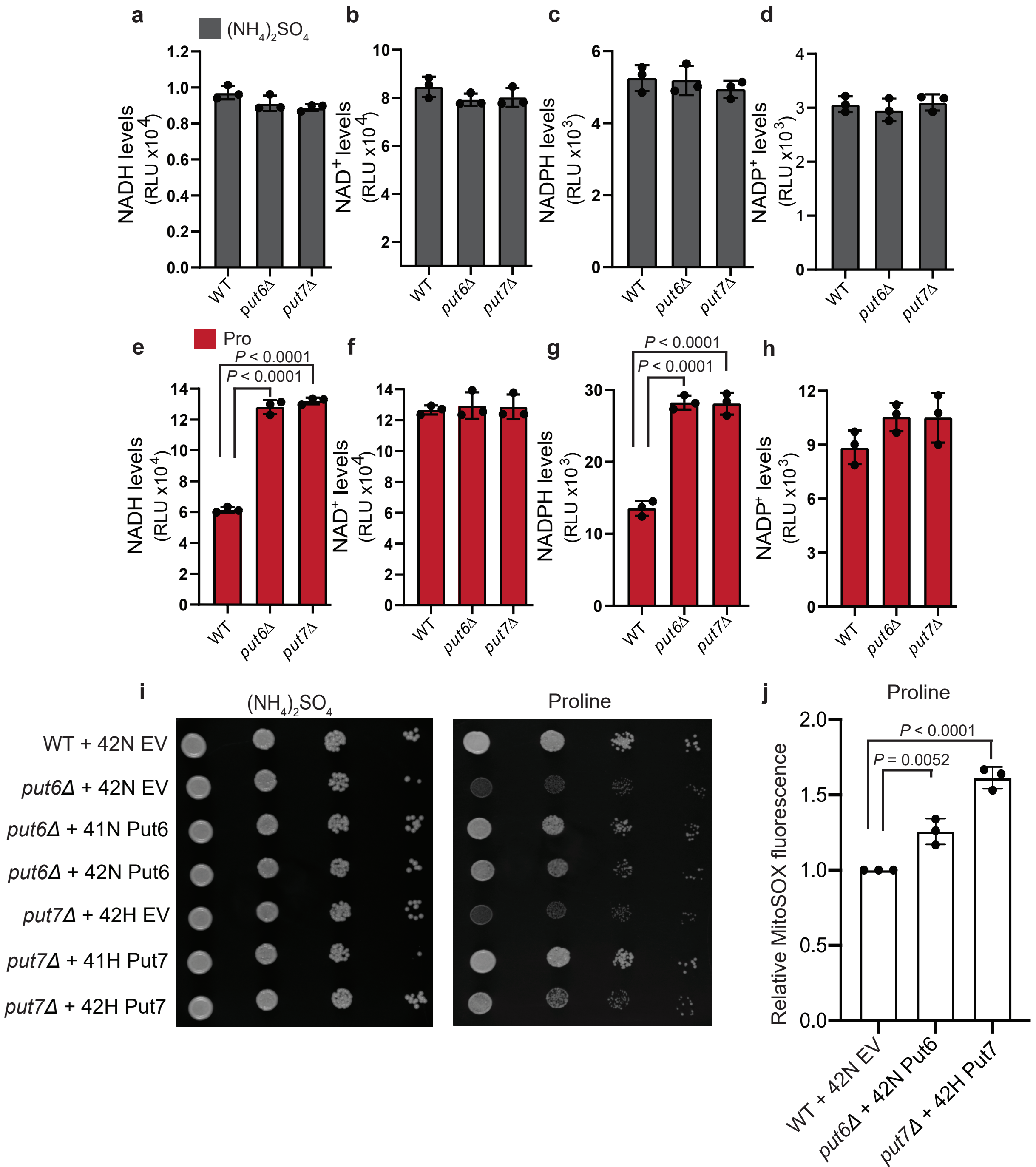

Supplementary Fig. 6: Loss or overexpression of Put6 and Put7 perturbs redox balance. (a-h) Levels of NADH, NAD+, NADPH and NADP+ in WT, *put6Δ* and *put7Δ* yeast cells grown on synthetic galactose media with ammonium sulfate (a-d) or proline (e-h) as the sole nitrogen source were measured using NAD/NADH-Glo™ and NADP/NADPH-Glo™ assays (Promega). Data were normalized to blank control and are expressed as mean ± SD (n=3 biologically independent experiments). **(i)** Ten-fold serial dilutions of indicated yeast strains harboring empty vector (EV) or Put6 and Put7 cloned in 41N, 41H (single copy) or 42N, 42H (multi-copy) vectors, were seeded on synthetic media plates containing proline as the sole nitrogen source and incubated at 30°C. Pictures were taken after 3 days of seeding. **(j)** Mitochondrial ROS measured using MitoSOX fluorescence in yeast cells grown in proline as the sole nitrogen source. Data were normalized to unstained control and are expressed as mean ± SD (n=3 biologically independent experiments) relative to WT. Statistical significance was assessed by one-way ANOVA with Tukey's multiple comparison test. The source data are provided as Source Data file.

Supplementary Table 1: *Saccharomyces cerevisiae* strains used in this study.

Saccharomyces cerevisiae strains	Source
BY4741 WT - *MATa, his3Δ1, leu2Δ0, met15Δ0, ura3Δ0*	Dr. Miriam L. Greenberg
BY4741 *put6*Δ - *MATa*, *his3Δ1, leu2Δ0, met15Δ0, ura3Δ0, put6Δ:: hphNT1*	This Study
BY4741 *put7*Δ - *MATa*, *his3Δ1, leu2Δ0, met15Δ0, ura3Δ0, put7Δ:: natMX4*	This Study
BY4741 *put6*Δ *put7*Δ - *MATa*, *his3Δ1, leu2Δ0, met15Δ0, ura3Δ0, put6:: hphNT1 put7Δ:: natMX4*	This Study
BY4742 WT - *MATα*, *his3Δ1, leu2Δ0, lys2Δ0, ura3Δ0*	Dr. Miriam L. Greenberg
BY4742 *put6Δ* - *MATα*, *his3Δ1, leu2Δ0, lys2Δ0, ura3Δ0, put6Δ:: hphNT1*	This Study
W303 WT- *MAT*a, *leu2-3,112 trp1-1, can1-100, ura3-1, ade2-1, his3-11,15*	Dr. Miriam L. Greenberg
W303 *put6Δ* - *MAT*a, *leu2-3,112 trp1-1, can1-100, ura3-1, ade2-1, his3-11,15, put6Δ:: hphNT1*	This Study
ACY WT- *MATa, hoΔ::KanMX*	Dr. Amy A. Caudy
ACY *put6Δ- MATa, hoΔ::KanMX, put6Δ:: hphNT1*	This Study
ACY *put7Δ- MATa, hoΔ::KanMX, put7Δ::* natMX4	This Study
ACY *put6Δ put7Δ – MATa, hoΔ::KanMX, put6Δ:: hphNT1 put7Δ::* natMX4	This Study
ACY *put1Δ- MATa, hoΔ::KanMX, put1Δ::hphNT1*	This Study
CEN.PK *MATa*	Dr. Benjamin Tu
CEN.PK *MATa, put6Δ::G418*	This Study
CEN.PK *MATa, put7Δ:: natMX4*	This Study
CEN.PK *MATa, put6Δ::G418 put7Δ:: natMX4*	This Study

Supplementary Table 2: Plasmids used in this study.

Plasmids	Source
pRS416	Dr. Craig Kaplan
pRS41H	Dr. Craig Kaplan
pRS41N	Dr. Craig Kaplan
pRS42H	Dr. Craig Kaplan
pRS42N	Dr. Craig Kaplan
pRS416 Put6	This Study
pRS416 Put6-V5	This Study
pRS416 Put7	This Study
pRS416 Put7-V5	This Study
pRS416 Put7-HA	This Study
pRS41N Put6	This Study
pRS41N Put6-V5	This Study
pRS42N Put6-V5	This Study
pRS41H Put7	This Study
pRS41H Put7-V5	This Study
pRS42H Put7-V5	This Study
pRS41N Codon optimized MCUR1-V5 (contains endogenous *PUT6* promoter)	This Study
pRS41H Codon optimized MCUR1-V5 (contains endogenous *PUT6* promoter)	This Study

Supplementary Table 3: Antibodies used in this study.

Antibodies	Source	Identifier
Mouse monoclonal anti-V5	ThermoFisher	Cat# 46-0705
Rabbit polyclonal anti-V5	ThermoFisher	Cat# PA1-993
Mouse monoclonal anti-Pgk1	ThermoFisher	Cat# 459250
Mouse monoclonal anti-HA	Sigma-Aldrich	Cat# H9658
Rabbit monoclonal anti-HA	Abcam	Cat# ab182009
Mouse monoclonal anti-Cox1	Abcam	Cat# ab110270
Mouse monoclonal anti-Cox2	Abcam	Cat# ab110271
Mouse monoclonal anti-Cox4	Abcam	Cat# ab110272
Mouse monoclonal anti-Por1	Abcam	Cat# ab110326
Rabbit polyclonal anti-Coa6	Dr. Vishal M. Gohil	N/A
Rabbit anti-Sdh2	Dr. Dennis Winge	N/A
Mouse anti-Rip1	Dr. Vincenzo Zara	N/A
Rabbit anti-Tom70	Dr. Jan Brix	N/A
Rabbit anti-Tim50	Dr. Jan Brix	N/A
Rabbit anti-Tom44	Dr. Jan Brix	N/A

Supplementary Table 4: Primers used in this study

Primer Name	Sequence 5'→ 3'
FMP32 Del For	GTGAGCGTAATAAGTAAAGTAAGGTAGAACGGAATAAAATGCAGATTGTACTGAGAGTGC
FMP32 Del Rev	CAAGTACCAAGTCTAGCCCTTTTTCCTCGCTTGTCGCCACTACCTTACGCATCTGTGCGG
YLR283W Del For	TTTATGTATACATGTAAACTCTGAACATCTAATAGACTAATGCGTACGCTGCAGGTCGAC
YLR283W Del Rev	ACACACAATATACTTATGTACAGACAATATGTATGCTAATTATCGATGAATTCGAGCTCG
Put1 Del For	AACATCGCTACATAGTAATAACACTAACGCACGCTAGAAATGCGTACGCTGCAGGTCGAC
Put1 Del Rev	TGGTTTGTCTTTGAAATTGGAGTATATATTATAGTCCTCTCATCGATGAATTCGAGCTCG
Fmp32-V5 For	CCCCTGGAGCTCAACCACACATAGCGACACTAGACGC
Fmp32-V5 Rev	GGGGCCTCTAGATTACGTAGAATCGAGACCGAGGAGA
YLR283W For	CCCCGCGAGCTCGCGCGCGAGCTTAAGCCGGGCAAGC
YLR283W Rev	CCCTGCCTCGAGATCTATATCATGTTGATCTTGAGGGA
YLR283W(nostop) BamH1Rev	CCCTGCGGATCCATCTATATCATGTTGATCTTGAGGGA
pRS416 3HA Ecor1 Rev	CCCAAAGAATTCTGCAAGCCGCACCTGCATGCTTAAGC
YLR283W BamH1 (stop) Rev	CCCTGCGGATCCTTAATCTATATCATGTTGATCTTGAGGGA
MCUR1-V5 For	ATGCATGAGCTCAACCACACATAGCGACACTAGACGC
MCUR1-V5 Rev	CCCCGGTCTAGACTAGGTAGAGTCCAAACCTAAC
