# Supplementary material for: Yeast homologs of human MCUR1 regulate mitochondrial proline metabolism
Source: Nat Commun. 2020 Sep 25;11:4866. doi: 10.1038/s41467-020-18704-1 (PMC7519068; doi:10.1038/s41467-020-18704-1)
Supplement: Supplementary file 3 — Reporting Summary [file 41467_2020_18704_MOESM3_ESM.pdf]

nature research

Corresponding author(s): Vishal M. Gohil

Last updated by author(s): Aug 18, 2020

Reporting Summary

Nature Research wishes to improve the reproducibility of the work that we publish. This form provides structure for consistency and transparency in reporting. For further information on Nature Research policies, seeAuthors & Referees and theEditorial Policy Checklist .

Statistics

For all statistical analyses, confirm that the following items are present in the figure legend, table legend, main text, or Methods section.

n/a	Confirmed	
☐	☒	The exact sample size (*n*) for each experimental group/condition, given as a discrete number and unit of measurement
☐	☒	A statement on whether measurements were taken from distinct samples or whether the same sample was measured repeatedly
☐	☒	The statistical test(s) used AND whether they are one- or two-sided<br>*Only common tests should be described solely by name; describe more complex techniques in the Methods section.*
☒	☐	A description of all covariates tested
☒	☐	A description of any assumptions or corrections, such as tests of normality and adjustment for multiple comparisons
☐	☒	A full description of the statistical parameters including central tendency (e.g. means) or other basic estimates (e.g. regression coefficient) AND variation (e.g. standard deviation) or associated estimates of uncertainty (e.g. confidence intervals)
☐	☒	For null hypothesis testing, the test statistic (e.g. *F*, *t*, *r*) with confidence intervals, effect sizes, degrees of freedom and *P* value noted<br>*Give P values as exact values whenever suitable.*
☒	☐	For Bayesian analysis, information on the choice of priors and Markov chain Monte Carlo settings
☒	☐	For hierarchical and complex designs, identification of the appropriate level for tests and full reporting of outcomes
☒	☐	Estimates of effect sizes (e.g. Cohen's *d*, Pearson's *r*), indicating how they were calculated

Our web collection on statistics for biologists contains articles on many of the points above.

Software and code

Policy information about availability of computer code

Data collection	Proteomics data acquisition: XcaliburTM version 2.9.0.293<br>LC-MS/MS metabolomics data acquisition - Thermo XcaliburTM software version 4.0.27.19
Data analysis	- Western blot: ImageJ<br>- Graphs and statistical analysis: Graphpad Prism 7.0<br>- Computational proteomics: MaxQuant version 1.6.1.13<br>- Proteomics data analysis: Perseus version 1.5.4.2<br>- LC-MS/MS metabolic analysis: MZmine 2.36

For manuscripts utilizing custom algorithms or software that are central to the research but not yet described in published literature, software must be made available to editors/reviewers. We strongly encourage code deposition in a community repository (e.g. GitHub). See the Nature Research guidelines for submitting code & software for further information.

Data

Policy information about availability of data

All manuscripts must include a data availability statement. This statement should provide the following information, where applicable:
- Accession codes, unique identifiers, or web links for publicly available datasets
- A list of figures that have associated raw data
- A description of any restrictions on data availability

The raw data from this study are available in the source file. The mass spectrometry proteomics data have been deposited to the ProteomeXchange Consortium via the PRIDE partner repository with the dataset identifier PXD015476.

Field-specific reporting

Please select the one below that is the best fit for your research. If you are not sure, read the appropriate sections before making your selection.

☒ Life sciences ☐ Behavioural & social sciences ☐ Ecological, evolutionary & environmental sciences

For a reference copy of the document with all sections, see nature.com/documents/nr-reporting-summary-flat.pdf

Life sciences study design

All studies must disclose on these points even when the disclosure is negative.

Sample size	Atleast three biological replicates were used for each experimental condition. Biological replicates are defined as independent experiments performed on different biological samples on different days. The design was based on prior assay experience and similar experiments reported in the literature.
Data exclusions	No data were excluded from the analyses.
Replication	All three biological replicates were successful.
Randomization	Samples were processed in random order.
Blinding	NA

Reporting for specific materials, systems and methods

We require information from authors about some types of materials, experimental systems and methods used in many studies. Here, indicate whether each material, system or method listed is relevant to your study. If you are not sure if a list item applies to your research, read the appropriate section before selecting a response.

Materials & experimental systems

n/a	Involved in the study	
☐	☒	Antibodies
☐	☒	Eukaryotic cell lines
☒	☐	Palaeontology
☒	☐	Animals and other organisms
☒	☐	Human research participants
☒	☐	Clinical data

Methods

n/a	Involved in the study	
☒	☐	ChIP-seq
☐	☒	Flow cytometry
☒	☐	MRI-based neuroimaging

Antibodies

Antibodies used	Mouse monoclonal anti-V5 (ThermoFisher, catalog 46-0705, lot 1949337); Rabbit polyclonal anti-V5 (ThermoFisher, catalog PA1-993, lot SL259858); Mouse monoclonal anti-Pgk1 (ThermoFisher, catalog 459250, lot H4728); Mouse monoclonal anti-HA (Sigma-Aldrich, catalog H9658, lot 024M4773); Rabbit monoclonal anti-HA (Abcam, catalog ab182009, lot GR152227-11); Mouse monoclonal anti-Cox1 (Abcam, catalog ab110270, lot GR201584-1); Mouse monoclonal anti-Cox2 (Abcam, catalog ab110271, lot GR260352-1); Mouse monoclonal anti-Cox4 (Abcam, catalog ab110272, lot GR88671-1); Mouse monoclonal anti-Por1 (Abcam, catalog ab110326, lot GR157859-1), Rabbit polyclonal anti-Coa6 (Dr. Vishal Gohil, Texas A&M University, USA); Rabbit anti-Sdh2 (Dr. Dennis Winge, The University of Utah, USA); Mouse anti-Rip1 (Dr. Vincenzo Zara, University of Salento, Italy); Rabbit anti-Tom70, Rabbit anti-Tim50, Rabbit anti-Tim44 (Dr. Jan Brix, University of Freiburg, Germany).
Validation	All commercial antibodies had validation statements and results on the manufacturer's websites.

Eukaryotic cell lines

Policy information about cell lines

Cell line source(s)	BY4741 and BY4742 WT yeast strains were gifts from Dr. Miriam L. Greenberg, Wayne State University, USA; ACY WT yeast strain was gift from Dr. Amy A Caudy, University of Toronto, Canada; CEN.PK WT yeast strain was gift from Dr. Benjamin Tu UT Southwestern Medical Center, USA.
Authentication	Yeast strains were tested for expression of known markers.
Mycoplasma contamination	NA

Commonly misidentified lines (See ICLAC register)	NA

Flow Cytometry

Plots

Confirm that:

- [x] The axis labels state the marker and fluorochrome used (e.g. CD4-FITC).
- [x] The axis scales are clearly visible. Include numbers along axes only for bottom left plot of group (a 'group' is an analysis of identical markers).
- [ ] All plots are contour plots with outliers or pseudocolor plots.
- [x] A numerical value for number of cells or percentage (with statistics) is provided.

Methodology

Sample preparation	Yeast cells were processed according to the method section for experiment and analysis.
Instrument	BD AccuriTM C6 flow cytometer
Software	CFlow Plus
Cell population abundance	10 million cells per ml, of which 10,000 cells were analyzed.
Gating strategy	NA

- [ ] Tick this box to confirm that a figure exemplifying the gating strategy is provided in the Supplementary Information.
